# Diffusing Mn^4+^ into Dy^3+^ Doped SrAl_2_O_4_ for Full-Color Tunable Emissions

**DOI:** 10.3390/ma15228170

**Published:** 2022-11-17

**Authors:** Bao-gai Zhai, Meng Meng Chen, Yuan Ming Huang

**Affiliations:** School of Microelectronics and Control Engineering, Changzhou University, Changzhou 213164, China

**Keywords:** strontium aluminate, diffusion temperature, diffusion time, red emission of Mn^4+^, photoluminescence

## Abstract

Dy^3+^ and Mn^4+^ codoped SrAl_2_O_4_ (SrAl_2_O_4_:Dy^3+^,Mn^4+^) phosphors were obtained by diffusing Mn^4+^ ions into Dy^3+^-doped SrAl_2_O_4_ via the constant-source diffusion technique. The influences of diffusion temperature and diffusion time on the emissions of SrAl_2_O_4_:Dy^3+^,Mn^4+^ were investigated. It was found that: (i) efficient red emission peaking at 651 nm can be readily achieved for SrAl_2_O_4_:Dy^3+^ by simply diffusing Mn^4+^ into SrAl_2_O_4_:Dy^3+^ at 800 °C and above; (ii) the red emission of Mn^4+^ becomes dominant over the characteristic emissions of Dy^3+^ when the diffusion temperature is 900 °C or higher; and (iii) the intensity of the red emission of SrAl_2_O_4_:Dy^3+^,Mn^4+^ is far more sensitive to diffusion temperature than to diffusion time. Our results have demonstrated that full-color tunable emissions can be realized for SrAl_2_O_4_:Dy^3+^, Mn^4+^ by tuning the parameters of diffusion temperature and diffusion time, which opens up a space for realizing easy color control of Dy^3+^-doped inorganic materials.

## 1. Introduction

Being a member of rare earth-activated luminescent materials, Dy^3+^-doped SrAl_2_O_4_ (SrAl_2_O_4_:Dy^3+^) has attracted much attention because of its interesting emission features [1,2,3,4]. Under ultraviolet excitation, the photoluminescence (PL) of SrAl_2_O_4_:Dy^3+^ is composed of two parts: (i) a broad emission band peaking at about 400 nm due to the intrinsic defects in SrAl_2_O_4_ [5]; and (ii) three narrow emission bands of Dy^3+^ activators centered at around 482, 574 nm and 663 nm, which are due to the optical transitions from ^4^F_9/2_ to ^6^H_15/2_, ^6^H_13/2_, and ^6^H_11/2_, respectively [6,7,8,9,10,11]. In principle, SrAl_2_O_4_:Dy^3+^ can be developed into a color-tunable light-emitting phosphor for applications in the solid state lighting industry because it contains blue, yellow, and red emissions. Unfortunately, the red emission band of Dy^3+^ is too weak to adjust the colorimetric performance of SrAl_2_O_4_:Dy^3+^. Consequently, the realization of full-color emissions for SrAl_2_O_4_:Dy^3+^ seems to be an intractable problem unless a facile technique is developed to endow efficient red emissions to SrAl_2_O_4_:Dy^3+^.

Red to deep red emission of Mn^4+^ (3d^3^ electron configuration) provides a solution to this problem. The ground state (^4^A_2g_) and the lowest excited state (^2^E_g_) of Mn^4+^ arise from the t^3^_2g_ configuration; the spin forbidden ^2^E_g_→^4^A_2g_ emission generally consists of a sharp line and associated vibronic sidebands. Under ultraviolet or blue irradiation, deep red emissions are observed for a number of Mn^4+^-doped inorganic hosts [12,13]; examples include the red emissions for Mn^4+^-doped SrAl_2_O_4_ (653 nm) [14], Sr_4_Al_14_O_25_ (662 nm) [15], Mg_2_TiO_4_ (665 nm) [16,17], CaAl_12_O_19_ (656 nm) [18], and CaAl_2_O_4_ (658 nm) [19]. Thus, codoping SrAl_2_O_4_:Dy^3+^ with Mn^4+^ seems to be a promising strategy to realize full-color tunable emissions for SrAl_2_O_4_:Dy^3+^. As documented in the literature, Chi et al. reported the red emission of Mn^4+^ in SrAl_2_O_4_ (around 660 nm) with a quenching concentration as low as 0.04 mol% [14]. Such a low quenching concentration poses stringent requirements on the doping technique, due to the fine control over the dose of Mn^4+^ in SrAl_2_O_4_:Dy^3+^,Mn^4+^. Making use of the advantages of the constant-source diffusion technique, our strategy in this work is to diffuse Mn^4+^ ions into SrAl_2_O_4_:Dy^3+^ at low doping levels by tuning the parameters of diffusion temperature and diffusion time. Our results demonstrate that intense red emission peaking at 651 nm can be achieved by diffusing Mn^4+^ into SrAl_2_O_4_:Dy^3+^ at 800ºC and higher, yielding full-color tunable emissions for Dy^3+^ and Mn^4+^ codoped SrAl_2_O_4_ (SrAl_2_O_4_:Dy^3+^,Mn^4+^).

## 2. Experimental Section

### 2.1. Preparation of SrAl_2_O_4_:Dy^3+^,Mn^4+^

The first step was to synthesize SrAl_2_O_4_:Dy^3+^. Using the sol-gel combustion technique, SrAl_2_O_4_:Dy^3+^ was synthesized by controlling the doping level of Dy^3+^ at 1.6 mol% [2,3,4]. Analytical grade reagents strontium nitrate (Sr(NO_3_)_2_), aluminum nitrate nonahydrate (Al(NO_3_)_3_·9H_2_O), urea, and boric acid (H_3_BO_3_) were purchased from a local chemical supplier, i.e., Sinopharm Chemical Reagents Co., Ltd. (Shanghai, China). Dysprosium oxide (Dy_2_O_3_) provided the source of Dy dopant, and the purity of Dy_2_O_3_ was 4 N. Under stirring with a magnetic bar, Al(NO_3_)_3_·9H_2_O (0.4 mol), Sr(NO_3_)_2_ (0.2 mol), H_3_BO_3_ (0.02 mol), urea (6.0 mol), and Dy_2_O_3_ (0.0016 mol) were dissolved in 600 mL of deionized water. A transparent solution was obtained after the mixture was vigorously stirred for 4 h in a glass beaker. After being aged at room temperature for two weeks, the solution could be used for the sol-gel combustion. Urea and boric acid in the solution functioned as the fuel and flux, respectively. Alumina crucibles were utilized as the containers for the sol-gel combustion; the volume capacity of each alumina crucible was 50 mL. After being filled with 25 mL of the aged solution, the alumina crucible was transferred into a box furnace where the sol-gel combustion reaction took place. The temperature in the box furnace was kept at 700 °C before the solution-containing crucible was added. During the combustion, the temperature in the furnace was elevated to about 820 °C, but the temperature in the flame could reach 1300 °C. After the sol-gel combustion, white powders were collected. According to the molar ratio of Dy^3+^ ions to Sr^2+^ ions in the solution, the nominal doping concentration of Dy^3+^ in SrAl_2_O_4_ was determined to be 1.6 mol%.

The second step was to yield SrAl_2_O_4_:Dy^3+^,Mn^4+^ by diffusing Mn^4+^ into SrAl_2_O_4_:Dy^3+^ via the constant-source diffusion technique. Cylindrical corundum crucibles (ϕ40 mm) with a height of 35 mm were employed as the vessel for diffusion. A thin layer of MnO_2_ (about 1 μm in thickness) was deposited onto the bottom of the crucible for constant-source diffusion. SrAl_2_O_4_:Dy^3+^ powders were stored in the MnO_2_-coated crucible, which was transferred into an air filled tubular furnace for Mn^4+^ diffusion. The diffusion was carried out under air atmosphere. The doses of Mn^4+^ in SrAl_2_O_4_:Dy^3+^,Mn^4+^ were controlled by tuning the diffusion temperature and diffusion time.

### 2.2. Phase, Morphology, and Elemental Composition of SrAl_2_O_4_:Dy^3+^,Mn^4+^

The phase of SrAl_2_O_4_:Dy^3+^,Mn^4+^ phosphors was determined by their X-ray diffraction (XRD) profiles. The XRD profiles of SrAl_2_O_4_:Dy^3+^,Mn^4+^ phosphors were recorded on an X-ray diffractometer (D/max 2500 PC, Rigaku Corporation, Akishima, Japan). The wavelength of the Cu Kα radiation was 0.15405 nm. The morphology of SrAl_2_O_4_:Dy^3+^,Mn^4+^ phosphors was characterized with a scanning electron microscope (SEM) (model S-4800, Hitachi, Tokyo, Japan). The elemental composition of SrAl_2_O_4_:Dy^3+^,Mn^4+^ phosphors was provided by the energy dispersive X-ray (EDX) spectrum of the synthesized phosphor. The nanostructures and crystal lattice of SrAl_2_O_4_:Dy^3+^,Mn^4+^ were investigated on a transmission electron microscope (TEM) (model JEOL JEM-2100, Japan Electronics Corp, Akishima, Japan). Samples for TEM analysis were prepared by dispersing the particles of the phosphor in ethanol. After being excited in an ultrasonic cleaner for 10 min, a drop of the suspension was dried on a carbon-coated copper grid.

To determine the valence states of dopants Mn^4+^ and Dy^3+^ in SrAl_2_O_4_:Dy^3+^,Mn^4+^, we performed X-ray photoelectron spectroscopy (XPS) analysis on an Escalab 250Xi spectrophotometer (Thermo Scientific, Waltham, MA, USA). Coming from Al Kα radiation, the energy of the incident X-ray was 1486.6 eV for the XPS characterization. Samples were not cleaned and not sputtered before XPS characterization. All the samples were analyzed by the charge correction. The binding energy of the standard C–C bond of the calibrated XPS instrument was 284.8 eV.

### 2.3. PL Spectra of SrAl_2_O_4_:Dy^3+^,Mn^4+^

The steady-state PL spectra of SrAl_2_O_4_:Dy^3+^,Mn^4+^ phosphors were acquired with a spectrophotometer (Tianjin Gangdong Ltd., Tianjin, China). A helium-cadmium laser (Kimmon Electric Co., Ltd., Tokyo, Japan) provided the excitation source for the PL measurement. The emission wavelength of the laser radiation was 325 nm; the output power of the laser radiation was 13 mW. Each PL spectrum was taken at room temperature.

### 2.4. PL Decay Curves of SrAl_2_O_4_:Dy^3+^,Mn^4+^

On a picosecond fluorescence lifetime spectrometer (LifeSpec II, Edinburgh Instruments, Edinburgh, UK), the PL decay curves of intrinsic defect-related emissions of SrAl_2_O_4_:Dy^3+^ before Mn^4+^ diffusion were measured at room temperature. The time correlated single photon counting technique was applied in the photon detection. A picosecond pulsed light-emitting diode provided the pulsed excitation. The excitation wavelength of the pulsed laser was 320 nm. The pulse width of the picosecond pulsed light-emitting diode was about 860 ps. For the PL decay curve, the detection wavelength was 400 nm, and the pulse repetition rate of the light source was fixed at 10 MHz, corresponding to a pulse every 100 ns. Details on the PL decay characterization could be found elsewhere [20,21]. In contrast, the PL decay curve of the SrAl_2_O_4_:Dy^3+^ after Mn^4+^ diffusion was measured at room temperature on a transient-state fluorescence spectrometer (FS5, Edinburgh Instruments, Edinburgh, UK). The PL decay curve of Mn^4+^ emission was recorded at 651 nm for SrAl_2_O_4_:Dy^3+^ after Mn^4+^ diffusion at 1000 °C. A pulsed Xenon lamp was employed to provide the pulsed excitation.

## 3. Results and Discussions

### 3.1. Phase and Morphology of SrAl_2_O_4_:Dy^3+^,Mn^4+^

**Figure 1:**
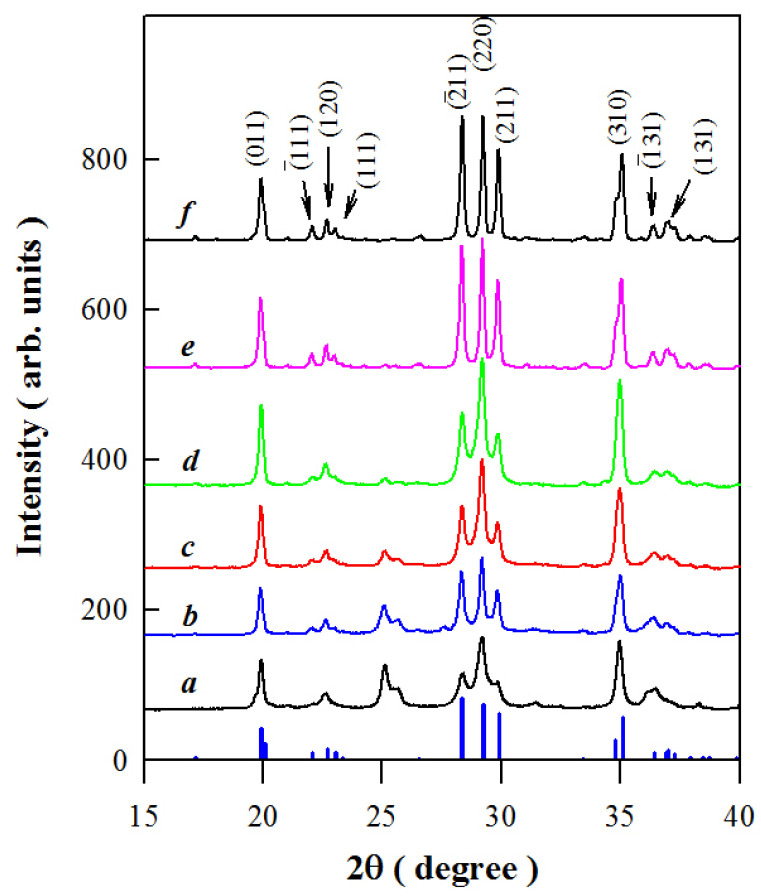
XRD curves of the SrAl_2_O_4_:Dy^3+^ before Mn^4+^ diffusion (*a*) and after Mn^4+^ diffusion at temperatures of 600 °C (*b*), 700 °C (*c*), 800 °C (*d*), 900 °C (*e*), and 1000 °C (*f*). The duration of each diffusion is 4 h. The doping concentration of Dy^3+^ is 1.6 mol%. The standard diffraction data of monoclinic SrAl_2_O_4_ (JCPDS card no. 34-0379) are shown at the bottom of the figure as vertical bars for comparison.

Figure 1 represents the X-ray diffraction (XRD) curves of SrAl_2_O_4_:Dy^3+^ before Mn^4+^ diffusion (*a*) and after Mn^4+^ diffusion at temperatures of 600 °C (*b*), 700 °C (*c*), 800 °C (*d*), 900 °C (*e*), and 1000 °C (*f*). The duration of each diffusion was 4 h. The diffraction data of standard monoclinic SrAl_2_O_4_ (JCPDS card no. 34-0379) are shown at the bottom of Figure 1 for comparison. Evidently, pure phase SrAl_2_O_4_ is formed when the diffusion temperatures are 900 and 1000 °C [2,3,4,5]. In contrast, a secondary phase is formed in SrAl_2_O_4_ before Mn^4+^ diffusion (curve *a*) and after Mn^4+^ diffusion at 600, 700, and 800 °C (curves *b*–*d*), due to the presence of a diffraction peak at 2θ = 25°. This phenomenon can be attributed to the formation of an aluminoborate complex in the combustion synthesis due to the addition of boric acid. Boric acid, which is used as a fluxing agent in promoting the formation of the required crystalline phase, can also behave as one of the precursor materials for the formation of aluminoborate complexes. The formed aluminoborate complexes can be decomposed and evaporated after annealing at high temperatures. This is the reason why the diffraction peak at 2θ = 25° gradually loses its intensity when the diffusion temperature is elevated from 600 to 800 °C.

Monoclinic SrAl_2_O_4_ has a stuffed tridymite structure with the space group P2_1_ and Z = 4. As described in our previous work, monoclinic SrAl_2_O_4_ has a three-dimensional network of corner-sharing [AlO_4_] tetrahedra with channels present in the *a*- and *c*-directions. Two crystallographically different Sr^2+^ sites are present in the unit cell of SrAl_2_O_4_, and these Sr^2+^ ions are located along the channels [5]. Therefore, it is easy for Dy^3+^ and Mn^4+^ to dope SrAl_2_O_4_. Depending on their coordination numbers (CN), the effective ionic radii of cations vary to some extent. In the case of site occupancy, the effective ionic radii of Sr^2+^, Dy^3+^, Al^3+^, and Mn^4+^ are 0.145 (CN = 9), 0.122 (CN = 9), 0.053 (CN = 4), and 0.053 nm (CN = 4), respectively [22]. Due to their comparable ionic sizes, dopants Dy^3+^ and Mn^4+^ tend to replace the Sr^2+^ and Al^3+^ sites in the lattice of SrAl_2_O_4_, respectively.

**Figure 2:**
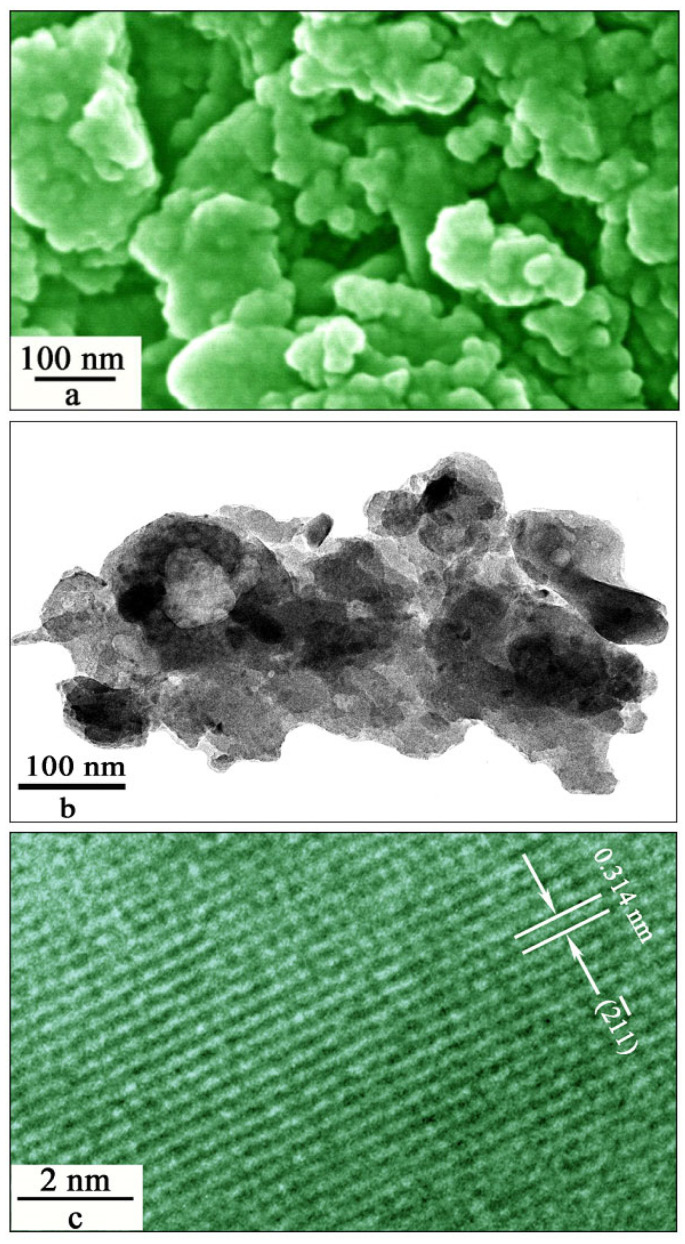
SEM micrograph (**a**), low-resolution TEM micrograph (**b**), high-resolution TEM micrograph (**c**) of SrAl_2_O_4_:Dy^3+^ after Mn^4+^ diffusion at 800 °C for 4 h. The doping concentration of Dy^3+^ is 1.6 mol%.

Figure 2 displays the SEM micrograph (a), low-magnification TEM micrograph (b), and high-resolution TEM micrograph (c) of SrAl_2_O_4_:Dy^3+^ after Mn^4+^ diffusion at 800 °C for 4 h. As shown in Figure 2a, some of the particles are as small as 20 nm in diameter, but they are prone to forming large aggregates because of their high surface energy. As shown in Figure 2b, the aggregate consists of a large number of nanoparticles with their dimensions up to 80 nm. Our previous work has evidenced the formation of a number of SrAl_2_O_4_ nanocrystals in a single aggregate [5]. The high-resolution TEM micrograph in Figure 2c illustrates the crystal lattices of one SrAl_2_O_4_:Dy^3+^,Mn^4+^ nanoparticle. The spacing between two adjacent planes in Figure 2c is found to be 0.314 nm, which is in good agreement with the distance between two adjacent ($\bar{2}$11) crystallographic planes of monoclinic SrAl_2_O_4_. Thus, the micrographs in Figure 2 demonstrate that the synthesized SrAl_2_O_4_:Dy^3+^, Mn^4+^ exhibits good crystallinity.

### 3.2. EDX Spectrum of SrAl_2_O_4_:Dy^3+^,Mn^4+^

**Figure 3:**
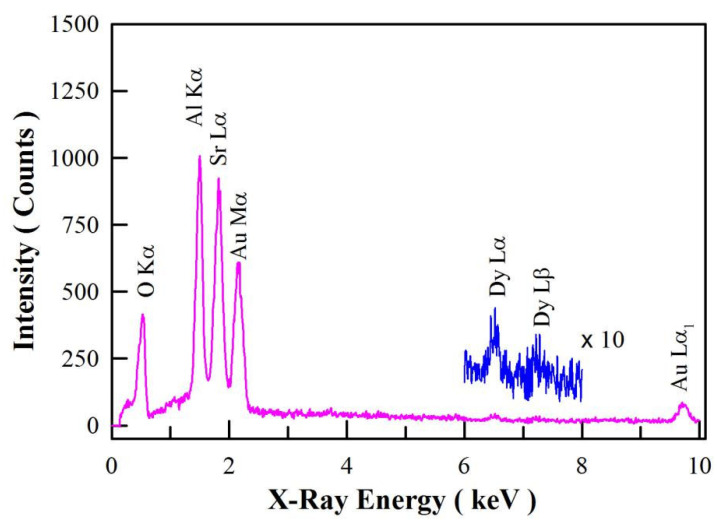
EDX spectrum of SrAl_2_O_4_:Dy^3+^ (1.6 mol%) after Mn^4+^ diffusion at 800 °C for 4 h.

Figure 3 illustrates the EDX spectrum of SrAl_2_O_4_:Dy^3+^ after Mn^4+^ diffusion at 800 °C for 4 h. As mentioned in the experimental section, the doping concentration of Dy^3+^ is 1.6 mol%. As can be seen in Figure 3, the characteristic X-ray emissions of O(Kα), Al(Kα), and Sr(Lα_1_) are located at 0.525, 1.486, and 1.806 keV, respectively. In the meantime, the characteristic emissions of Dy(Lα_1_) and Dy(Lβ_1_) can be identified at 6.495 and 7.248 keV, respectively. Additionally, the X-ray emissions of Au(Mα_1_) and Au(Lα_1_) are located at 2.122 and 9.713 keV, respectively. However, element Mn is not detected with the EDX because the doping concentration is very low, which is outside the detection limit of the EDX facility. The detection limit of our EDX is about 1 wt. % for element Mn. As previously described, the Au element in the specimen is introduced in the process of Au sputtering for the convenience of SEM characterization. As expected, the EDX spectrum of SrAl_2_O_4_:Dy^3+^,Mn^4+^ verifies the presence of elements Al, Sr, O, and Dy in the phosphor.

### 3.3. XPS Spectrum of SrAl_2_O_4_:Dy^3+^,Mn^4+^

**Figure 4:**
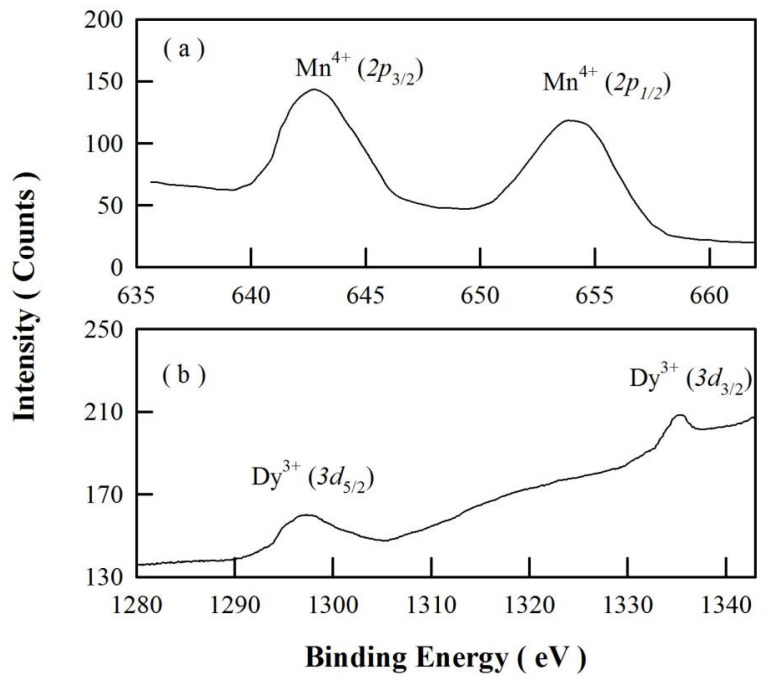
High-resolution XPS spectra of Mn 2p (**a**) and Dy 3d (**b**) in SrAl_2_O_4_:Dy^3+^ after Mn^4+^ diffusion at 900 °C for 4 h.

The oxidation states of Mn and Dy in SrAl_2_O_4_:Dy^3+^,Mn^4+^ are examined with the XPS analysis. Figure 4 represents the high-resolution XPS spectra of Mn 2p (a) and Dy 3d (b) in SrAl_2_O_4_:Dy^3+^,Mn^4+^. Mn^4+^ ions are diffused into SrAl_2_O_4_:Dy^3+^ at 900 °C for 4 h. As can be seen in Figure 4a, the peaks of Mn 2p_3/2_ and Mn 2p_1/2_ are located at about 642.2 and 653.8 eV, respectively. These binding energies indicate that the oxidation state of Mn ion is 4+. Figure 4b demonstrates that the peaks of the spin-orbit component (3d_5/2_ and 3d_3/2_) of Dy^3+^ are located at approximately 1297.6 and 1335.1 eV, respectively. The high-resolution XPS spectrum of O 1s in SrAl_2_O_4_:Dy^3+^,Mn^4+^ nanocrystals is shown in Figure S1. Peaking at 531.78 eV, the XPS spectral profile of O1s can be deconvoluted into several components. 

### 3.4. PL Spectra of SrAl_2_O_4_:Dy^3+^ after Mn^4+^ Diffusion at Different Temperatures

**Figure 5:**
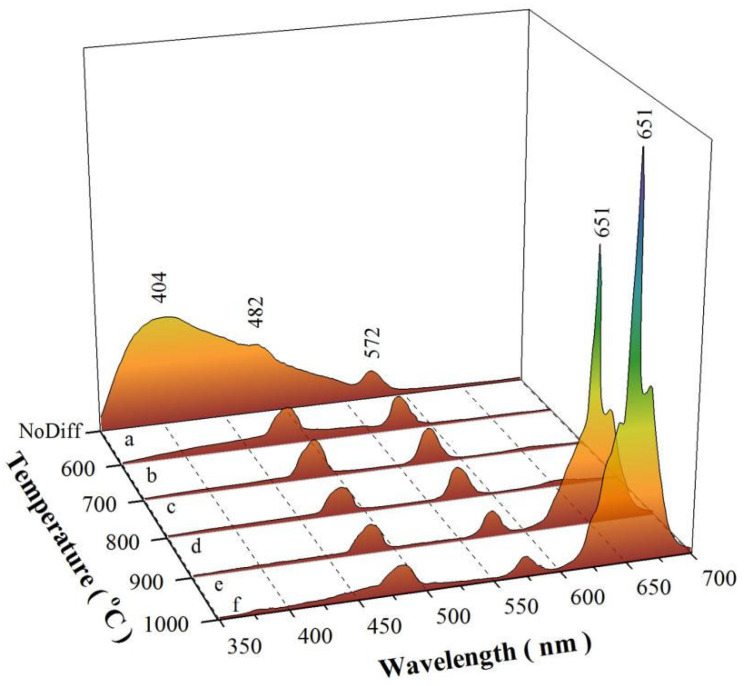
PL spectra of SrAl_2_O_4_:Dy^3+^ before Mn^4+^ diffusion (a) and after Mn^4+^ diffusion at 600 °C (b), 700 °C (c), 800 °C (d), 900 °C (e), and 1000 °C (f). The duration of each diffusion is 4 h.

Figure 5 represents the PL spectra of SrAl_2_O_4_:Dy^3+^ before Mn^4+^ diffusion (a) and after Mn^4+^ diffusion at 600 °C (b), 700 °C (c), 800 °C (d), 900 °C (e), and 1000 °C (f). The diffusion time is 4 h for each sample. As can be seen in Figure 5a, the PL spectrum of the SrAl_2_O_4_:Dy^3+^ before Mn^4+^ diffusion consists of a broad PL band peaking at about 400 nm, and two characteristic emission bands of Dy^3+^ ions peaking at 482 and 572 nm, respectively. The broad emission band of SrAl_2_O_4_:Dy^3+^ comes from intrinsic defects (namely oxygen and/or strontium vacancies) in the lattice of SrAl_2_O_4_ [5]. The narrow emission bands peaking at 482 and 572 nm are due to the ^4^F_9/2_ → ^6^H_15/2_ and ^4^F_9/2_ → ^6^H_13/2_ transitions of Dy^3+^ [2,3,4,6]. Before Mn^4+^ diffusion, the broad emission band of SrAl_2_O_4_:Dy^3+^ is very strong. Conversely, the two narrow emission bands centered at 482 and 572 nm are very weak. No red emission can be detected in the range of 600–700 nm for SrAl_2_O_4_:Dy^3+^ before Mn^4+^ diffusion. Thus, the perception color of the emissions of SrAl_2_O_4_:Dy^3+^ before Mn^4+^ diffusion is blue (Figure S2). Actually, quite similar intrinsic defect-related emissions have been recorded for a large number of aluminates, such as undoped CaAl_2_O_4_ [20,23], Dy^3+^-doped BaAl_2_O_4_ [21], Tb^3+^-doped CaAl_2_O_4_ [24], and Tb^3+^-doped SrAl_2_O_4_ [25]. At high annealing temperatures, oxygen atoms in air can migrate into the crystal lattice to repair the defects with the result of a decline in the population density of oxygen vacancies in SrAl_2_O_4_:Dy^3+^. Therefore, the intrinsic defect-related emissions should be weakened or even have disappeared after air annealing at high temperatures. Indeed, the broad emission band of SrAl_2_O_4_:Dy^3+^ disappears when the diffusion temperature is elevated to 600 °C and higher, as shown in Figure 5b–f.

The most striking feature in Figure 5 is that the red emission peaking at 651 nm gradually gains intensity as the diffusion temperature increases from 600 to 1000 °C. Figure S3 illustrates the PL spectra of SrAl_2_O_4_:Dy^3+^ after Mn^4+^ diffusion at 800 °C (a), 900 °C (b), and 1000 °C (c) for 4 h. The inset in Figure S3 shows the zoomed section of the PL spectra of SrAl_2_O_4_:Dy^3+^,Mn^4+^ in the range of 620–700 nm. Quantitative analysis shows that the intensities of the red PL band are about 3%, 9%, and 30% that of the narrow blue PL band of Dy^3+^, peaking at 482 nm when SrAl_2_O_4_:Dy^3+^ is subject to Mn^4+^ diffusion at 600, 700, and 800 °C, respectively. Surprisingly, the red PL band of SrAl_2_O_4_:Dy^3+^,Mn^4+^ becomes dominant in intensity when the diffusion temperature is beyond 800 °C. As shown by the PL spectra *e* and *f* in Figure 5, the peak of the red PL band is located at 651 nm, which is in good agreement with the deep red emission of Mn^4+^-doped SrAl_2_O_4_ [14]. The integrated PL intensities of the red emission in PL spectra *e* and *f* are about 11.7 and 20.9 fold stronger than that of the narrow blue PL band of Dy^3+^ peaking at 482 nm. Thus, the PL spectra *e* and *f* verify that strong red emission can be achieved via Mn^4+^ diffusion into SrAl_2_O_4_:Dy^3+^ at diffusion temperatures higher than 800 °C. The PL quantum efficiency is a key parameter for the quantification of luminescent processes in phosphors. Characterized by Quantaurus-QY (Hamamatsu, Japan), the PL quantum yield values of these compounds are found to vary in the range of 27–60%, depending on the diffusion temperature and the diffusion time. The PL quantum yield values of commercial phosphors are higher than 75%. For example, the PL quantum yield of Y_3_Al_5_O_12_:Ce^3+^ is known to be around 97%, and the green silicate Ba_2_SiO_4_:Eu^2+^ shows a PL quantum yield of about 79%. Compared to the high PL quantum yield values of these commercial phosphors, the PL quantum yield values of SrAl_2_O_4_:Dy^3+^,Mn^4+^ are quite low.

Once Mn^4+^ is diffused into SrAl_2_O_4_:Dy^3+^, a set of energy levels are introduced into the bandgap of SrAl_2_O_4_. As documented in the literature, the ground state and the excited state of Mn^4+^ can be denoted as ^2^E_g_ and ^4^A_2g_, respectively [15], and the red emission peaking at 651 nm is associated with the ^2^E_g_ → ^4^A_2g_ transition of Mn^4+^. One might wonder how it would be possible to make the red emission much stronger than the characteristic emissions of Dy^3+^ in SrAl_2_O_4_:Dy^3+^,Mn^4+^. The answer lies in the much higher dose of Mn^4+^ in SrAl_2_O_4_:Dy^3+^,Mn^4^ at higher diffusion temperatures. The diffusion coefficient is known to depend on temperature. The diffusion coefficient in solids at different temperatures is found to be well predicted by the Arrhenius equation:

(1) $$D=D_{0}\exp\left(-\frac{E_{A}}{kT}\right)$$

where *E_A_* is the activation energy for diffusion (in eV), *D_0_* is the diffusion coefficient at infinite temperature (in cm^2^/s), *D* is the diffusion coefficient (in cm^2^/s), *k* is the Boltzmann constant, *T* is the absolute temperature (in K). The dose, which is the total amount of a dopant diffused into a solid, of a constant-source diffusion can be expressed as:

(2) $$Q=\frac{2N_{0}}{\sqrt{\pi }}\sqrt{Dt}$$

where *Q* is the dose in the host (atoms/cm), *N_0_* is the surface concentration (atoms/cm^2^), *D* is the diffusion coefficient (cm^2^/s), and *t* is the diffusion time (s). In the constant source diffusion, the dose increases as a function of diffusion temperature and diffusion time. Equations (1) and (2) predict that the dose quickly increases with the diffusion temperature for given values of *E_A_* and *t*. The activation energy for Mn^4+^ diffusion in SrA_l2_O_4_ has not yet been reported. An estimation of the activation energy for Mn^4+^ diffusion in SrAl_2_O_4_ becomes a choice. As documented in the literature, de Biasi and Grillo reported that the activation energies of 266 kJ/mol (2.76 eV) for the diffusion of Mn^2+^ in CaO and 203 kJ/mol (2.10 eV) for the diffusion of Mn^2+^ in MgO [26]. Additionally, Portavoce et al. reported that the activation energy for Mn in monocrystalline Ge is 2.37 eV [27]. After having considered the typical activation energies of As in polysilicon (3.9 eV), B in polysilicon (2.4–2.5 eV), B in SiO_2_ (2.38–3.53 eV), Ga in SiO_2_ (4.17 eV), and As in SiO_2_ (3.7–4.7 eV), it seems reasonable to assume that the activation energy for Mn^4+^ diffusion in SrAl_2_O_4_ is in the range of 2–5 eV. Assuming that *E_A_* takes the value of 3.0 eV, the doses of Mn^4+^ diffused into SrAl_2_O_4_ at 700, 800, 900, and 1000 °C are 7.8, 41, 163, and 523 times as large, respectively, as the dose of Mn^4+^ diffused into SrAl_2_O_4_ at 600 °C (Figure S4). The quick increase in the dose of Mn^4+^ at high diffusion temperature makes the red emission of Mn^4+^ dominant over the characteristic emissions of Dy^3+^ in SrAl_2_O_4_:Dy^3+^,Mn^4^. As a result, full-color tunable emissions are realized for SrAl_2_O_4_:Dy^3+^,Mn^4^ due to the complementary red emission of Mn^4+^. Rather than doping the host with red-emitting rare earth ions, such as Eu^3+^ (^5^D_0_→^7^F_2_ at 612 nm) [11,28], Sm^3+^ (^4^G_5/2_→^6^H_7/2_ at 605 nm) [29], or Pr^3+^ (^3^P_1_→^3^F_3_ at 642 nm) [30], diffusing Mn^4+^ into SrAl_2_O_4_:Dy^3+^ makes the red emission cost effective.

### 3.5. Emission Colors of SrAl_2_O_4_:Dy^3+^,Mn^4+^

**Figure 6:**
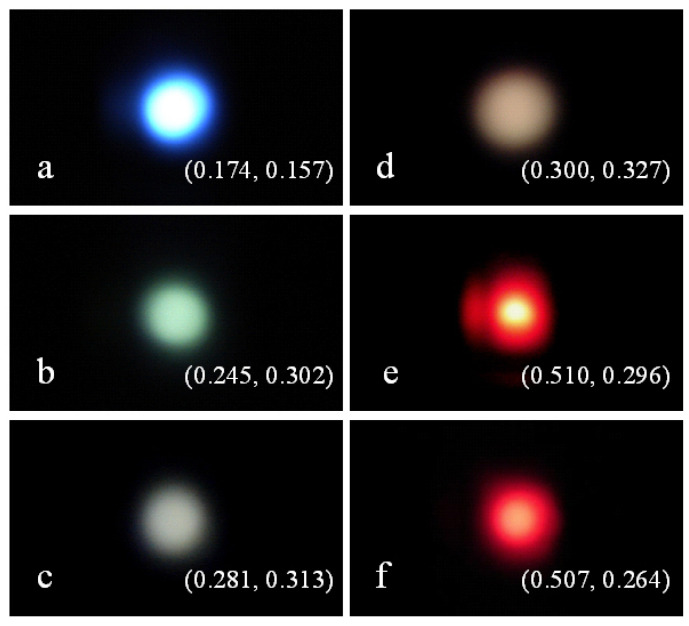
Photographs of the PL from SrAl_2_O_4_:Dy^3+^ before Mn^4+^ diffusion (**a**) and after Mn^4+^ diffusion at temperatures of 600 °C (**b**), 700 °C (**c**), 800 °C (**d**), 900 °C (**e**), and 1000 °C (**f**). The diffusion time is 4 h.

Along with the characteristic emissions of Dy^3+^ peaking at 482 nm (blue) and 572 nm (yellow), the emergence of the red emission of Mn^4+^ makes SrAl_2_O_4_:Dy^3+^,Mn^4+^ suitable for multi-color emissions. Color coordinates, which are important parameters to quantitatively describe the emission color for luminescent materials, can be calculated from their PL spectral data [31,32]. Figure 6 shows photographs of the light-emitting SrAl_2_O_4_:Dy^3+^ before Mn^4+^ diffusion (a) and after Mn^4+^ diffusion at 600 °C (b), 700 °C (c), 800 °C (d), 900 °C (e), and 1000 °C (f). The chromaticity coordinates are marked onto the corresponding photographs. Before Mn^4+^ diffusion, the PL color is blue with the chromaticity coordinates of (0.174, 0.157) for SrAl_2_O_4_:Dy^3+^, as shown in Figure 6a. When the diffusion temperature arises from 600 to 800 °C, the emission color of SrAl_2_O_4_:Dy^3+^,Mn^4+^ evolves from bluish green to white due to the weakened blue emission of the host at high diffusion temperature, as shown in Figure 6b–d. Interestingly, the emission color dramatically changes when SrAl_2_O_4_:Dy^3+^ is subject to Mn^4+^ diffusion at a temperature higher than 800 °C. As illustrated in Figure 6e,f, the emission colors of SrAl_2_O_4_:Dy^3+^,Mn^4+^ are pink and purplish red when the diffusion temperatures are 900 and 1000 °C, respectively. The dramatic changes in emission color are due to the enhanced red emission of Mn^4+^. The CIE chromaticity diagram of the emissions of SrAl_2_O_4_:Dy^3+^ before Mn^4+^ diffusion (a) and after Mn^4+^ diffusion at temperatures of 600 °C (b), 700 °C (c), 800 °C (d), 900 °C (e), and 1000 °C (f) is shown in Figure S5. It is clear that full-color tunable emissions can be achieved for SrAl_2_O_4_:Dy^3+^,Mn^4+^ by tuning the diffusion temperature of Mn^4+^.

### 3.6. PL Spectra of SrAl_2_O_4_:Dy^3+^ after Mn^4+^ Diffusion for Different Times

**Figure 7:**
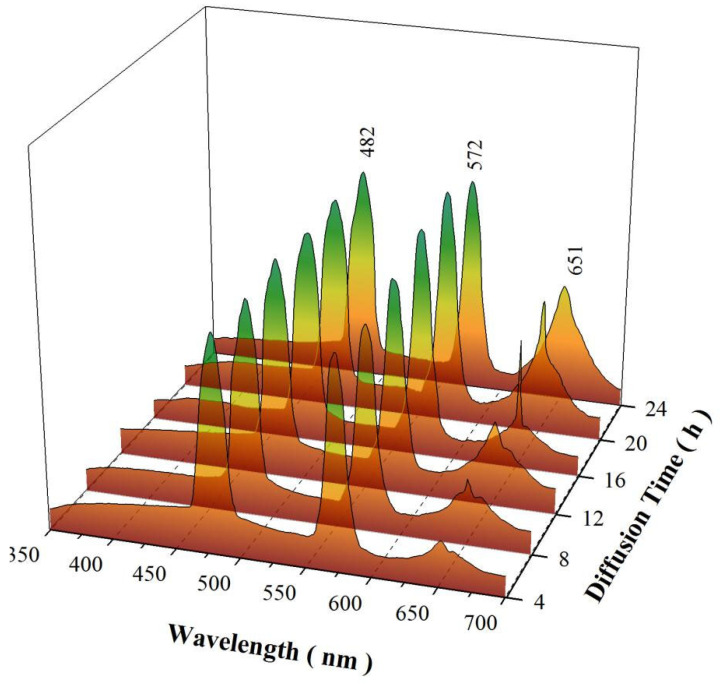
PL spectra of SrAl_2_O_4_:Dy^3+^ (1.6 mol%) after Mn^4+^ diffusion at 800 °C for different times: 4 h, 8 h, 12 h, 16 h, 20 h, and 24 h.

According to Equation (2), the dose of the constant-source diffusion increases with the diffusion time when the diffusion temperature is fixed. Therefore, the dose of Mn^4+^ in SrAl_2_O_4_:Dy^3+^,Mn^4+^ can be tuned by the diffusion time. Figure 7 depicts the PL spectra of SrAl_2_O_4_:Dy^3+^ after Mn^4+^ diffusion at 800 °C for different times: 4 h, 8 h, 12 h, 16 h, 20 h, and 24 h. It can be seen in Figure 7 that each PL spectrum consists of one blue emission band centered at 482 nm, one yellow emission band centered at 572 nm, and one red band peaking at about 651 nm. Obviously, the sharp emission peak at 651 nm can be ascribed to the ^2^E_g_→^4^A_2g_ transition of tetrahedrally-coordinated Mn^4+^ [16]. The quenching concentration of Mn^4+^ in SrAl_2_O_4_ was reported to be 0.04 mol% [14]. The red emission in Figure 7 increases slowly with the diffusion time, indicating that the concentration of Mn^4+^ in SrAl_2_O_4_:Dy^3+^,Mn^4+^ can be well controlled before reaching the quenching concentration. According to Equation (2), the doses of Mn^4+^ diffused into SrAl_2_O_4_:Dy^3+^ at 800 °C for 8, 12, 16, 20, and 24 h are 1.4, 1.7, 2.0, 2.2, and 2.5 times as large as the dose of Mn^4+^ diffused into SrAl_2_O_4_:Dy^3+^ at 800 °C for 4 h. Thus, it is understandable that the diffusion temperature, rather than the diffusion time, is more effective in enhancing the red emission of SrAl_2_O_4_:Dy^3+^,Mn^4+^. Obviously, diffusing Mn^4+^ into SrAl_2_O_4_:Dy^3+^ by the constant-source diffusion technique exhibits advantages because tuning the parameters of diffusion temperature and diffusion time can finely control the doping concentration at low doping levels. 

Thermal diffusion of Mn^4+^ into SrAl_2_O_4_:Dy^3+^ has potential industrial sense because it modifies the PL properties of SrAl_2_O_4_:Dy^3+^ phosphor with the introduction of red emissions of Mn^4+^ ions. The diffusion time of 4 h seems quite long when compared to the diffusion times of B and P doping silicon in advanced semiconductor fabrication sectors. Optimizing the diffusion time and diffusion temperature is a key procedure for the application of SrAl_2_O_4_:Dy^3+^,Mn^4+^ as phosphors in the solid state lighting industry.

### 3.7. PL Decays of SrAl_2_O_4_:Dy^3+^,Mn^4+^

**Figure 8:**
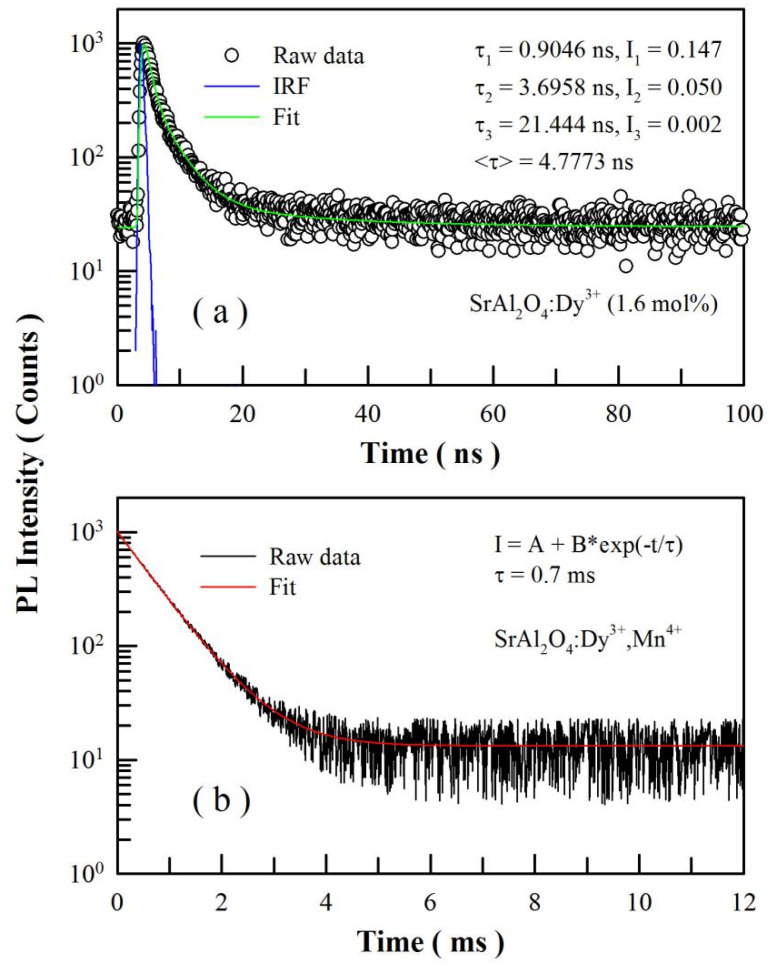
(**a**) PL decay curve of SrAl_2_O_4_:Dy^3+^ before Mn^4+^ diffusion; the detection wavelengths is 400 nm. (**b**) PL decay curve of CaAl_2_O_4_:Dy^3+^ after Mn^4+^ diffusion at 1000 °C for 4 h; the detection wavelength is 651 nm. The excitation wavelength is 320 nm.

Figure 8a represents the PL decay curve of SrAl_2_O_4_:Dy^3+^ before Mn^4+^ diffusion. The excitation wavelength for this decay curve is 320; the detection wavelength is 400 nm. The instrument response function is shown in Figure 8a (in blue) for the decay curve. The exponential reconvolution of the raw data is represented by the green solid curve in Figure 8a. Our analysis reveals that this PL decay curve can be best described by a tri-exponential decay model with three largely different time constants. The fitting parameters of the PL decay are listed in Figure 8a. On one hand, the three time constants are largely different, suggesting the presence of three distinctly different channels in the phosphor for radiative recombination. On the other hand, the averaged lifetime of the PL decay is 4.78 ns, which provides complementary information about the intrinsic defect-related emissions of SrAl_2_O_4_.

Figure 8b represents the PL decay of SrAl_2_O_4_:Dy^3+^ after Mn^4+^ diffusion at 1000 °C for 4 h. The excitation wavelength for this decay curve is 320; the detection wavelength is 651 nm. The exponential reconvolution of the raw data in Figure 8b is represented by the red solid curve. Clearly, the PL decay curve in Figure 8b is well described by a mono-exponential decay model with a time constant of 0.7 ms. As documented in the literature, Cao et al. reported that the lifetime of Mn^4+^ emission in Mn^4+^-doped CaAl_2_O_4_ decreased from 1.35 to 0.94 ms as the concentration of Mn^4+^ increased from 0.2 to 1.6 mol% [19]. Sun et al. reported that the lifetime of Mn^4+^ emissions in Mn^4+^-doped Ba_2_GdTaO_6_ was around 0.3 ms [33]. Apparently, the PL lifetime of Mn^4+^ emissions in SrAl_2_O_4_:Dy^3+^,Mn^4+^ is in line with those of Mn^4+^ emissions in a large number of Mn^4+^-doped inorganic materials [19,33,34,35,36]. The lifetime of Mn^4+^ emission of SrAl_2_O_4_:Dy^3+^,Mn^4+^ is about five orders of magnitude higher than that of the intrinsic defect-related emissions of SrAl_2_O_4_:Dy^3+^, meaning that the red emission is caused by the parity-forbidden d-d transitions of the Mn^4+^ ions.

The PL decay time of the defect-related blue emission band is 4.78 ns, which is unusually fast when compared to the PL decay time of the defect-related emission in some complex oxides, such as tungstates and molybdates. For example, the PL decay times of PbMoO_4_ and ZnWO_4_ crystals are of the order of tens and hundreds of microseconds [37]. We tried to measure the decay of this broad blue band in microsecond or even millisecond scales, but no obvious changes in the PL decay time were found for our sol-gel-derived SrAl_2_O_4_:Dy^3+^,Mn^4+^ when the time scales of the PL decay measurements were extended to 200, 500, 1000, 2000, 5000, and 50,000 ns, respectively. It is known the PL decay time of a phosphor depends on the non-radiative recombination rates in the phosphor. The higher the non-radiative recombination rate, the shorter the PL decay time will be. When compared to PbMoO_4_ and ZnWO_4_ single crystals, the sol-gel-derived SrAl_2_O_4_:Dy^3+^,Mn^4+^ nanocrystals have a much higher density of oxygen vacancies, which in turn enhances the non-radiative recombination rate. Therefore, the high density of oxygen vacancies in the sol-gel-derived SrAl_2_O_4_:Dy^3+^,Mn^4+^ nanocrystals is responsible for the short PL decay time. Such an interpretation is supported by the averaged PL decay times of several nanoseconds for ZnWO_4_ nanocrystals [38,39], ZnMoO_4_ nanocrystals [40], zinc molybdenum oxide hydrate nanocrystals [41], and pentazinc dimolybdate pentahydrate [42]. In particular, our previous work revealed that the averaged PL decay time of ZnWO_4_ nanocrystals can be extended from 2.25 to 1770 ns when the population density of oxygen vacancies in ZnWO_4_ nanocrystals is decreased via thermal annealing in air [43].

## 4. Conclusions

Employing a constant-source diffusion technique, SrAl_2_O_4_:Dy^3+^,Mn^4+^ phosphors were obtained by diffusing Mn^4+^ ions into SrAl_2_O_4_:Dy^3+^. The influences of diffusion temperature and diffusion time on the red emission were investigated. It was found that: (i) peaking at 651 nm, the red emission of SrAl_2_O_4_:Dy^3+^,Mn^4+^ became dominant over the characteristic emissions of Dy^3+^ when the diffusion temperature was higher than 800 °C; (ii) the red emission intensity of SrAl_2_O_4_:Dy^3+^,Mn^4+^ was far more sensitive to diffusion temperature than to diffusion time; and (iii) full-color tunable emissions were realized for SrAl_2_O_4_:Dy^3+^,Mn^4+^ by constant source diffusion. Owing to the high degree of control over the dose of Mn^4+^ in SrAl_2_O_4_:Dy^3+^, diffusing Mn^4+^ into SrAl_2_O_4_:Dy^3+^ provided a unique opportunity to prepare full-color tunable phosphors. This approach could be the basis for convenient color control of Dy^3+^-doped materials by controlling the red emission intensity of Mn^4+^ in the host.

## Data Availability

Data are available upon request.

## Supplementary Materials

The following supporting information can be downloaded at: https://www.mdpi.com/article/10.3390/ma15228170/s1. Figure S1. High-resolution XPS spectrum of O 1s in SrAl_2_O_4_:Dy^3+^,Mn^4+^ nanocrystals. Figure S2. PL photograph of SrAl_2_O_4_:Dy^3+^ before Mn^4+^ diffusion. Doping concentration of Dy^3+^ is 1.6 mol% Excitation wavelength is 325 nm. Figure S3. PL spectra of SrAl_2_O_4_:Dy^3+^ after Mn^4+^ diffusion at 800 °C (a), 900 °C (b), and 1000 °C (c) for 4 h. Inset: zoomed section of the PL spectra of SrAl_2_O_4_:Dy^3+^, Mn^4+^ in the range of 620–700 nm. Figure S4. Normalized dose of Mn^4+^ in SrAl_2_O_4_:Dy^3+^ as a function of diffusion temperature for a given value of *E_A_*. Figure S5. CIE chromaticity diagram of the emissions from SrAl_2_O_4_:Dy^3+^ before Mn^4+^ diffusion (a) and after Mn^4+^ diffusion at 600 °C (b), 700 °C (c), 800 °C (d), 900 °C (e) and 1000 °C (f). The diffusion time is 4 h.
