# Supplementary material for: Diffusing Mn4+ into Dy3+ Doped SrAl2O4 for Full-Color Tunable Emissions
Source: Materials (Basel). 2022 Nov 17;15(22):8170. doi: 10.3390/ma15228170 (PMC9696492; doi:10.3390/ma15228170)
Supplement: Supplementary file 1 [file materials-15-08170-s001.zip › materials-1973984-supplementary.pdf]

Supplementary Materials

Diffusing Mn^{4+} into Dy^{3+} doped $SrAl_2O_4$ for full-color tunable emissions

Bao–gai Zhai, Meng Meng Chen, Yuan Ming Huang*

School of Microelectronics and Control Engineering, Changzhou University, Changzhou 213164, China

Figure S1. High-resolution XPS spectrum of O 1s in $SrAl_2O_4$:Dy^{3+},Mn^{4+} nanocrystals.

Figure S2. PL photograph of $SrAl_2O_4$:Dy^{3+} before Mn^{4+} diffusion. Doping concentration of Dy^{3+} is 1.6 mol%. Excitation wavelength is 325 nm.

Figure S3. PL spectra of $SrAl_2O_4$:Dy^{3+} after Mn^{4+} diffusion at 800ºC (a), 900ºC (b), and 1000ºC (c) for 4 h. Inset: zoomed section of the PL spectra of $SrAl_2O_4$:Dy^{3+}, Mn^{4+} in the range of 620–700 nm.

Figure S4. Normalized dose of Mn^{4+} in $SrAl_2O_4$:Dy^{3+} as a function of diffusion temperature for a given value of E_A.

Figure S5. CIE chromaticity diagram of the emissions from $SrAl_2O_4$:Dy^{3+} before Mn^{4+} diffusion (a) and after Mn^{4+} diffusion at 600ºC (b), 700ºC (c), 800ºC (d), 900ºC (e) and 1000ºC (f). The diffusion time is 4 h.

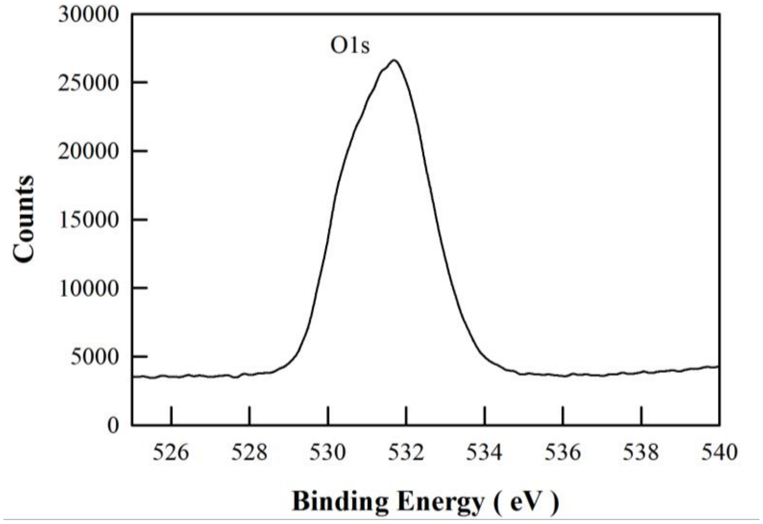

Figure S1. High-resolution XPS spectrum of O 1s in $SrAl_2O_4$:Dy^{3+},Mn^{4+} nanocrystals.

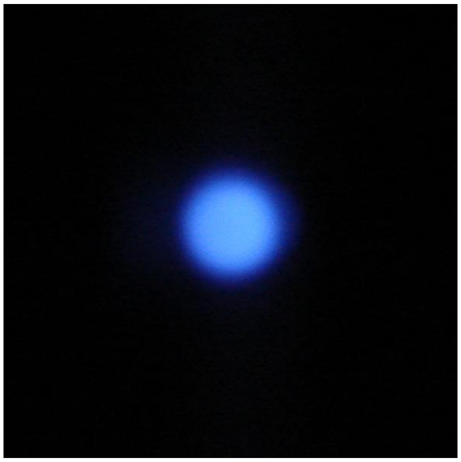

Figure S2. PL photograph of $SrAl_2O_4$:Dy^{3+} before Mn^{4+} diffusion. Doping concentration of Dy^{3+} is 1.6 mol%. Excitation wavelength is 325 nm.

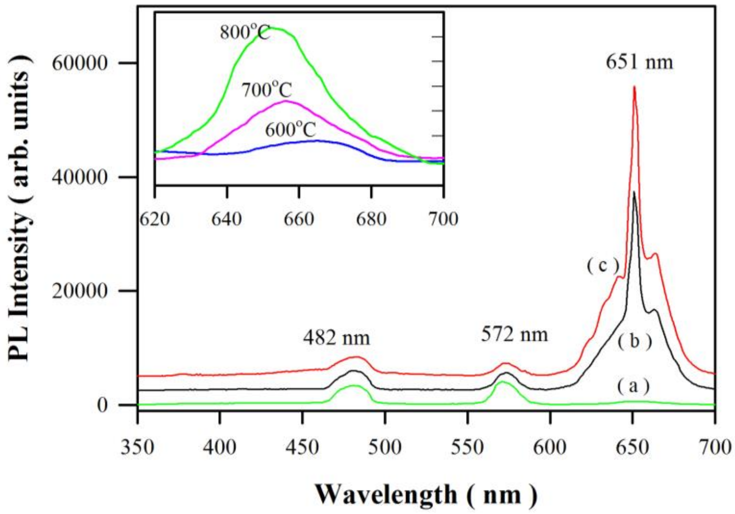

Figure S3. PL spectra of $SrAl_2O_4:Dy^{3+}$ after Mn^{4+} diffusion at 800ºC (a), 900ºC (b), and 1000ºC (c) for 4 h. Inset: zoomed section of the PL spectra of $SrAl_2O_4:Dy^{3+}$, Mn^{4+} in the range of 620–700 nm.

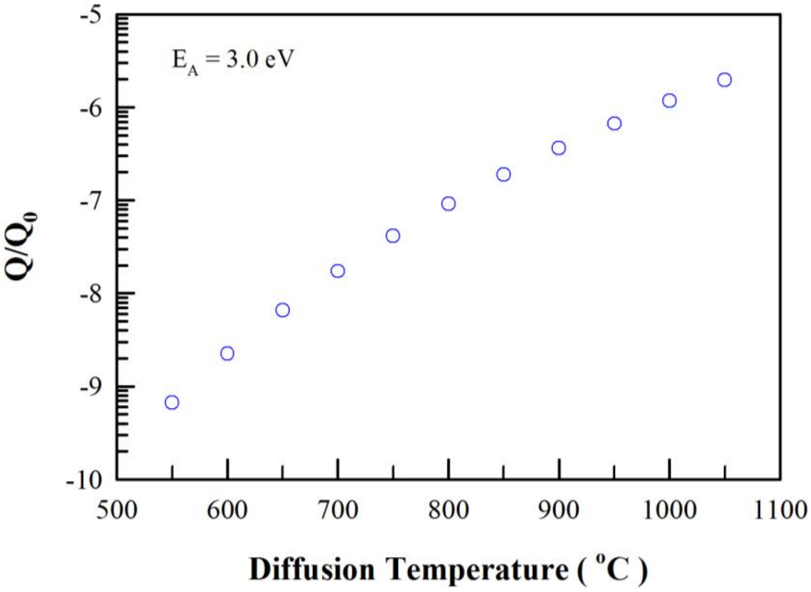

Figure S4. Normalized dose of Mn^{4+} in $SrAl_2O_4:Dy^{3+}$ as a function of diffusion temperature for a given value of E_A.

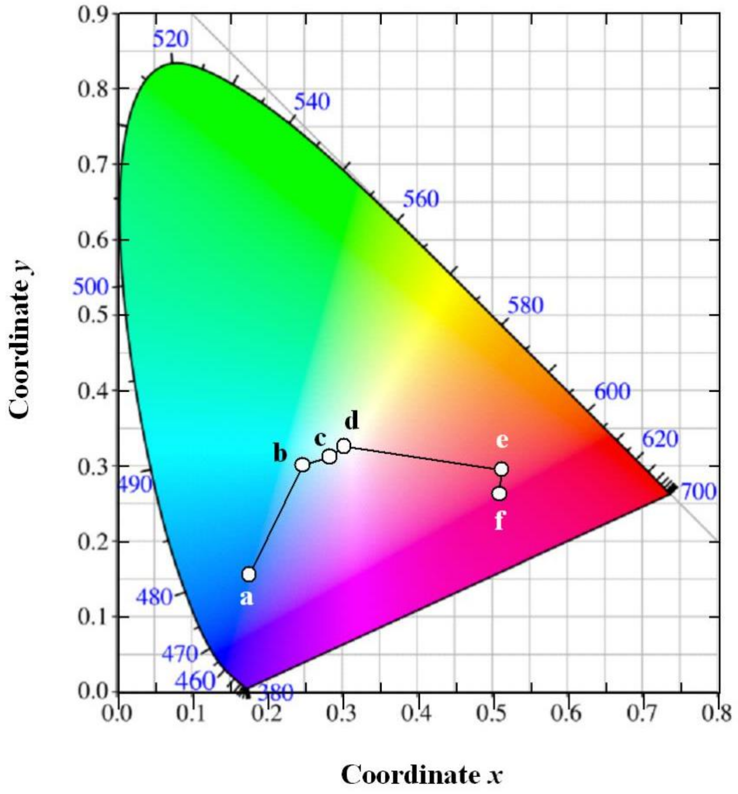

Figure S5. CIE chromaticity diagram of the emissions from $SrAl_2O_4:Dy^{3+}$ before Mn^{4+} diffusion (a) and after Mn^{4+} diffusion at 600ºC (b), 700ºC (c), 800ºC (d), 900ºC (e) and 1000ºC (f). The diffusion time is 4 h.
